# Comparative Proteomic Profiling of Tumor-Associated Proteins in Human Gastric Cancer Cells Treated with Pectolinarigenin

**DOI:** 10.3390/nu10111596

**Published:** 2018-10-30

**Authors:** Ho Jeong Lee, Venu Venkatarame Gowda Saralamma, Seong Min Kim, Sang Eun Ha, Preethi Vetrivel, Eun Hee Kim, Snag Joon Lee, Jeong Doo Heo, Shailima Rampogu, Keun Woo Lee, Gon Sup Kim

**Affiliations:** 1 Research Institute of Life Science and College of Veterinary Medicine, Gyeongsang National University, 501 Jinju-daero, Jinju 52828, Korea; hojeong.lee@kitox.re.kr (H.J.L.); gowdavenu27@gmail.com (V.V.G.S.); ksm4234@naver.com (S.M.K.); sangdis2@naver.com (S.E.H.); preethivetrivel05@gmail.com (P.V.); 2 Gyeongnam Department of Environment Toxicology and Chemistry, Toxicity Screening Research Center, Korea Institute of Toxicology, 17 Jegok-gil, Munsan-eup, Jinju 52834, Korea; sjlee@kitox.re.kr; 3 Department of Nursing Science, International University of Korea, 965 Dongbu-ro, Munsan-eup, Jinju 52833, Korea; iuknurse@nate.com; 4 Division of Life Sciences, Division of Applied Life Science (BK21 Plus Program), Plant Molecular Biology and Biotechnology Research Center (PMBBRC), Research Institute of Natural Science (RINS), Gyeongsang National University, Jinju 52828, Korea; shailima.rampogu@gmail.com (S.R.); kwlee@gnu.ac.kr (K.W.L.)

**Keywords:** gastric cancer, pectolinarigenin, 2-DE, DDX4, LRSAM1

## Abstract

Pectolinarigenin (PEC), a natural flavonoid that is present in citrus fruits, has been reported to exhibit antitumor effects in several cancers. Though the mechanism of PEC-induced cytotoxicity effects has been documented, the proteomic changes that are associated with the cellular response to this flavonoid are poorly understood in gastric cancer cells. In this study, a comparative proteomic analysis was performed to identify proteins associated with PEC-induced cell death in two human gastric cancer cell lines: AGS and MKN-28. Two-dimensional gel electrophoresis (2-DE) revealed a total of 29 and 56 protein spots with significant alteration were screened in AGS and MKN-28 cells respectively. In total, 13 (AGS) and 39 (MKN28) proteins were successfully identified by mass spectrometry from the differential spots and they are known to be involved in signal transduction, apoptosis, transcription and translation, cell structural organization, and metabolism, as is consistent with multiple effects of PEC on tumor cells. Notably, novel target proteins like Probable ATP-dependent RNA helicase DDX4 (DDX4) and E3 ubiquitin-protein ligase LRSAM1 (LRSAM1) along with the commonly differential expressed proteins on both the cell lines that are treated with PEC were confirmed by immunoblotting. The DDX4 accelerates cell cycle progression by abrogating the G2 checkpoint when overexpressed in cancer cells, while the aberrant expression of LRSAM1 may be involved in the cancer pathology. Thus, proteomic analysis provides vital information about target proteins that are important for PEC-induced cell death in gastric cancer cells.

## 1. Introduction

Cancer is one of the major public health concerns in both developing and developed countries [1]. It is considered as the second leading cause of death worldwide with gastric cancer identified as one of the most common recorded cancer cases in the world. Worldwide, Korea is recognized to have the highest cases of gastric cancer related deaths [2,3]. Even though the mortality rate of gastric cancer patients were reduced in Korea, gastric cancer related mortality remains the second leading cause of death worldwide and it is still the most prevalent cancer in Eastern Asia [1,3,4]. Over the past few years, analytical techniques from single genetic analysis to proteomic studies have been expanded and cancer treatments have been focused to be developed at a rapid rate. The 2-DE-based quantitative proteomic strategy provides a dynamic tool to interpret protein expression patterns globally and provides comprehensive analysis of changes in expression levels in terms of cellular localization, protein-protein interactions, and protein function. Since proteins play vital functions at both the cellular and molecular level, many researchers are interested to know the key proteins that represent the activity of the disease, such as cancer. Proteomic studies could lead to the molecular characterization of cellular event associated with cancer progression, signaling and response to drug treatment. From a therapeutic point of view, most of the drugs in cancer therapy targets proteins, not nucleic acids [5,6]. Proteome analysis has been applied in the investigation of various types of cancer studies (in-vitro and in-vivo), including gastric cancer for the discovery of drug targets and also to find new biomarkers of cancer [7]. The anti-cancer drug-regulated proteins identified by proteomic analysis can be further characterized as credible drug targets and effectors. The global analysis of protein modification will contribute imperative information to depict the mechanisms of drug action.

Phytochemicals derived from plants, fungi, and marine organisms has a long tradition in medicine [8]. Flavonoids are such group of bioactive polyphenol compounds, present in fruits, vegetables, and oriental plant that exhibit distinct biological activities, like anti-cancer and anti-inflammatory effect [9,10,11]. Pectolinarigenin (PEC) is one of the flavonoids compounds from our *Citrus platymamma* flavonoid extract, and it is also known to be present in enormous in *Cirsium* isolates [12,13]. PEC was found to synergistically stimulate apoptosis in MCF-7 breast cancer cells via the down regulation of Bcl2 expression [14]. In our previous study, PEC treatment showed an anti-cancer effect by inducing G2/M phase cell cycle arrest, autophagic, and apoptotic cell death in human gastric cancer cells by the down-regulation of PI3K/AKT/mTOR pathway [15]. In this study, for the comprehensive identification and characterization of functionally inflected proteins involved in PEC-induced cellular responses, we employed two-dimensional gel electrophoresis (2-DE) coupled with matrix-assisted laser desorption/ionization time-of-flight mass spectrometry (MALDI/TOF/MS) in AGS and MKN28 cells treated with PEC to epitomize the molecular mechanisms that are involved in PEC induced cell death. Several differential proteins that are involved in the regulation of Cell cycle, cellular growth, and apoptotic process in gastric cancer cells were identified to be regulated by PEC. The proteins professing an altered abundance after treatment with PEC may provide evidence for the future molecular research on the anti-cancer effect of PEC.

## 2. Materials and Methods

### 2.1. Chemicals and Reagent

The AGS and MKN28 human gastric cancer cells were obtained from the Korea Cell Line Bank (Seoul, Korea). RPMI-1640 medium, fetal bovine serum (FBS), and antibiotics (Penicillin/Streptomycin) were purchased from Gibco; Thermo Fisher Scientific, Inc. (Waltham, MA, USA). Pectolinarigenin were purchased from AdooQ (Irvine, CA, USA) 3-(4,5-Dimethylthiazol-2-yl)-2,5-diphenyltetrazolium bromide (MTT) was obtained from Sigma-Aldrich. Materials and chemicals used for electrophoresis were obtained from Bio-Rad Laboratories, Inc., (Hercules, CA, USA). Antibody to LRSAM1, DDX4, PI3KCB, and CIP2A were purchased from Cell Signaling Technology (Danvers, MA, USA). β-actin was purchased from Millipore (Billerica, MA, USA). Horseradish peroxidase (HRP)—conjugated goat anti-mouse IgG (ALX-211-205TS-C100) and anti-rabbit IgG (ADI-SAB-301-J) were purchased from Enzo life sciences.

### 2.2. Cell Viability Assay

The AGS cells and MKN28 cells (1 × 10^5^) were grown and maintained in RPMI-1640 medium supplemented with 1% penicillin/streptomycin and 10% heat-inactivated FBS in a humidified incubator with 5% CO_2_ in air 37 °C. The cells were seeded in 12-well plates and incubated overnight. The cells were subsequently treated with 0, 25, 50, 75, 100, 150, and 200 μM of PEC for 24 h. After incubation, to all wells 100 μL of 0.5 mg/mL MTT solution were added and incubated for 3 h at 37 °C in the dark. MTT solution containing media were removed and solubilized the formazan contained in the cells by the addition of 500 μL of dimethyl sulfoxide (DMSO), and the absorbance was measured at 540 nm using an enzyme-linked immunosorbent assay plate reader. The absorbance was positively correlated to the number of viable cells, so that cell viability was represented as the percentage of absorbance between treated and untreated cells.

### 2.3. Preparation of the Cellular Extract for 2-DE

Total proteins were extracted from the AGS and MKN28 cells in the PEC-treated and untreated (control) groups. Briefly, the cells were lysed with lysis buffer (2 M thiourea, 7 M urea, and 4% (*w*/*v*) CHAPS) on ice for 1 h after incubation with PEC. The lysates were then centrifuged at 14,000 RPM for 15 min at 4 °C, and collected supernatant. Proteins present in the supernatant was precipitated with 10% TCA (*v*/*v*) (1:1 ratio) incubated for 30 min at 4 °C. The protein samples were then washed twice with ice-cold 70% acetone twice and protein pellets were lyophilized in a lyophilizer dryer (SFDSM06, Samwon Freezing Engineering Co., Busan, Korea), and the protein pellets were dissolved in 200 μL of sample buffer and stored at 80 °C until further analysis. The concentration of protein was determined using the Pierce™ BCA protein assay kit (Thermo Scientific™, Waltham, MA, USA), in accordance with the manufacturer’s protocol.

### 2.4. Separation of Proteins by 2-DE and Image Analysis

For the first-dimension, an equal quantity (300 μg) of protein per sample were mixed with rehydration solution and loaded onto GE Healthcare Immobiline™ DryStrip Gels (18 cm, pH 4–7; Amersham Biosciences, Uppsala, Sweden) for first-dimensional isoelectric focusing (IEF) on an Ettan DALT II system (Amersham Biosciences). The focused strips were equilibrated twice for 15 min each time, first equilibration in 10 mg/mL dithiothreitol (DTT) and second one in 40 mg/mL iodoacetamide (IAA) prepared in an equilibration buffer containing 50 mM Tris–HCl (pH 8.8), 30% (*v*/*v*) glycerol, 6 M urea, and 2% (*w*/*v*) sodium dodecyl sulfate (SDS), which was followed by 12% second dimension sodium dodecyl sulfate-polyacrylamide gel electrophoresis (SDS-PAGE). The gels were stained with silver nitrate, as described previously [16] with slight modifications (Omit aldehyde in the fixative step), and three independent gels were used in triplicates. Briefly, gels were fixed with the fixation solution (50% ethanol and 5% acetic acid) and incubated for 30 min, washed once with 30% ethanol for 15 min. followed by three times with distilled water for 5 min each. Sensitized the gels with 0.02% Sodium Thiosulfate and gels were stained with silver nitrate (0.3%) in the dark for 25 min at room temperature. The gels were subsequently rinsed with water three times and developed with a developing solution (3% sodium carbonate, 0.02% sodium thiosulfate, and 0.05% formalin). For image analysis, gels were scanned and performed using Progenesis Samespots software (Nonlinear Dynamics, Newcastle, UK). The criterion of a differential expression between PEC-treated untreated AGS and MKN28 group of cells was a 1.5-fold change (*p* < 0.05) in spot volume between matched sets in triplicate.

### 2.5. Matrix-Assisted Laser Desorption/Ionization-Time of Flight Mass Spectrometry (MALDI-TOF MS) Mass Spectrometry Analysis

Selected differential protein spots were excised manually from the silver stained gels, and protein digestion was performed as defined previously with slight modifications [17]. Briefly, the excised gel pieces were washed destines water for 10 min three times and followed by washing with 100 μL 100 mM NH_4_HCO_3_ for 5 min, and then dehydrated in 100 μL of acetonitrile for 10 min. After being dried in a lyophilizer (SFDSM06, Samwon Freezing Engineering Co., Busan, Korea), the gel pieces were rehydrated in 5–10 μL of 50 mM NH_4_HCO_3_ containing 20 ng/μL trypsin (Promega Corporation, Madison, WI, USA) on ice for 45 min. After 45 min, the trypsin solution was replaced with 10–20 μL of 50 mM NH_4_HCO_3_ without trypsin, and digestion was carried out for a minimum of 16 h at 37 °C. Subsequently extracted by an addition of 10–20 μL of the extraction buffer, followed by an addition of 10–15 μL of acetonitrile, the supernatants were pooled. Peptide extracts were vacuum-dried in a lyophilizer and the extracts were re-dissolved in 1 μL of extraction buffer and 1 μL of matrix solution (α-acyano-4-hydroxycinnamic acid, HCCA) and targeted onto a MALDI-TOF plate and analyzed using a Voyager-DE STR mass spectrometer (Applied Biosystems, Franklin Lakes, NJ, USA), equipped with delay ion extraction. Mass Spectra were obtained over a mass range of ≥3000 Da.

### 2.6. Database Search for Protein Identification

Proteins were identified using the MS-Fit program in the ProteinProspector (http://www.prospector.ucsf.edu). The SwissProt database and peptide mass fingerprinting (PMF) data were used to determine matching proteins. For the database searches: Taxonomy, Homo sapiens (human); cleavage specificity, trypsin with one missed cleavage permitted; peptide tolerance of less than 100 ppm for the fragment ions; permitted modifications, fixed cysteine carbamidomethylation, variable oxidation of methionine, parameters were used. Protein MOWSE scores were considered statically significant (*p* < 0.05).

### 2.7. Western Blot Analysis

Immunoblotting was used to validate the differential expression of mass spectrometry identified proteins. Both the AGS and MKN28 cells (3 × 10^6^) were cultured in six-well plates and incubated with PEC (100 μM) for 24 h. Cells lysed with a lysis buffer [50 mM Tris-HCl (pH 8.0), 0.5% sodium deoxycholate, 1 mM EDTA, 150 mM NaCl, 0.1 sodium dodecyl sulfate (SDS), and 1% NP-40]. Protein concentration was determined using Pierce™ BCA protein assay kit (Thermo Scientific™, Waltham, MA, USA), in accordance with the manufacturer’s protocol. 20 μg of proteins were separated by 12% SDS-PAGE and transferred onto a PVDF membrane using the TE 77 Semi-Dry Transfer Unit (CE Healthcare Life Sciences, Buckinghamshire, UK). The blots were then blocked with 5% skimmed milk for 1 h at room temperature and then incubated with primary antibodies overnight (dilution 1:1000). Membranes were washed in TBS-T (3 × 10 min) and then probed with the appropriate horseradish peroxidase-coupled secondary antibody (dilution 1:2000) for 3 h at room temperature. The signal was visualized using Clarity™ ECL substrate reagent (Bio-Rad, Hercules, CA, USA) and quantified by densitometry while using the Image J (http://rsb.info.nih.gov) program. The densitometry readings of the bands were normalized to the expression of β-actin. The experiment was repeated three times.

### 2.8. Molecular Docking Studies

Molecular docking studies impart knowledge on the binding affinities between the protein and the ligand correspondingly, which determine the quintessential binding modes of a ligand [18]. For the current investigation, the ligand coordinates were drawn from the template and the key residues were marked for all the atoms around 10 Å. Correspondingly, the identified key residues were Tyr308, Lys310, Gln315, Gly335, Ser336, Gly337, Lys338, Thr339, and Asp605. The two-dimensional (2D) structures of PEC was imported from PubChem database and subsequently minimized with CHARMM force field by enabling the inbuilt Minimize Ligands module embedded in the DS in their 3D structures. For the docking studies, the CDOCKER protocol available with the DS was employed that operates by simulated annealing approach. Furthermore, each ligand was allowed to generate 30 confirmation and the best pose was selected based upon the highest -CDOCKER interaction energy retrieved from the largest cluster that has displayed intermolecular interactions with the key residues.

### 2.9. Protein-Protein Interaction & Gene Ontology (GO) Analysis

We investigated potential protein-protein interactions among selected genes by STRING (Search Tool for the Retrieval of Interacting Genes) database version 10.5 (http://string-db.org). STRING is web-based tool that readily provides uniquely comprehensive coverage and ease of access to both experimental as well as predicted interaction information. Gene ontology studies provided a brief description on the expressed proteins [19,20]. The gene expression profile of the expressed proteins was obtained using GENECODIS (http://genecodis.cnb.csic.es). Also, significantly enriched molecular pathways regulated by pectolinarigenin in AGS and MKN cells were identified.

### 2.10. Statistical Analysis

Experiments were performed three times by triplicate and results were represented as mean ± standard deviation (S.D.). Student’s *t*-test with using SPSS Version 10.0 for Windows (SPSS, Chicago, IL, USA) and one-way ANOVA test were employed for data analysis. The results are expressed as the mean ± standard deviation (SD) of at least three independent experiments. A *p* < 0.05 was considered as statistically significant.

## 3. Results

### 3.1. Pectolinarigenin (PEC) Reduced Cell Viability

**Figure 1:**
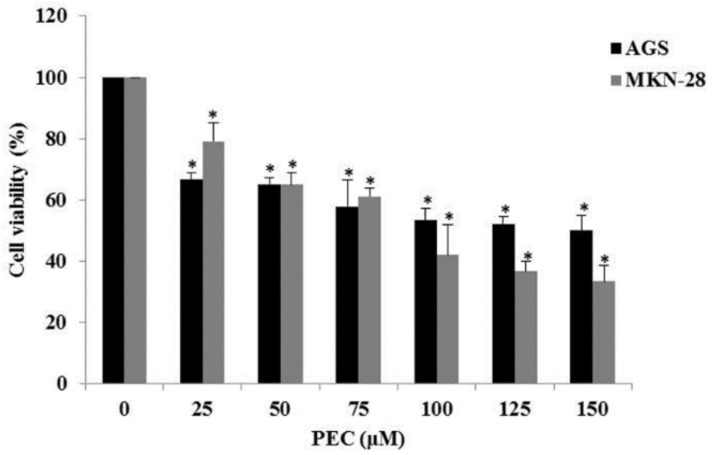
Pectolinarigenin (PEC) reduced cell viability of human gastric cancer cells. AGS and MKN28 human gastric cancer cells were incubated with concentration ranging from 0 to 150 μM of PEC for 24 h. Results are expressed as the mean ± standard deviation (SD) of at least three independent experiments. Statistical differences were analyzed with Student’s *t*-test (* *p* < 0.05 vs. control).

It has been reported that PEC was able to decrease the cell viability of AGS and MKN28 cell, human gastric cancer cells through the induction of cell cycle arrest, apoptosis, and autophagy. In this study, MTT assay was performed after treatment with PEC at various concentrations (0, 25, 50, 75, 100, and 150 μM) for 24 h (Figure 1) on AGS and MKN28 cells treated with PEC for cell viability. PEC reduced cell viability in a dose-dependent manner on both AGS and MKN28 cells when compared to the control group (DMSO only). The 50% inhibitory concentration (IC_50_) value was approximately attained at 100 μM (AGS IC_50_; 124.79 μM, MKN28 IC_50_; 96.88 μM) on both AGS and MKN28 cells (*p* < 0.05 for the PEC-treated group as compared with the control). Henceforth, we used 0 and 100 μM concentration of PEC for the subsequent experiments in both the cells.

### 3.2. 2-DE Analysis and Protein Identification by MALDI/TOF-MS

**Figure 2:**
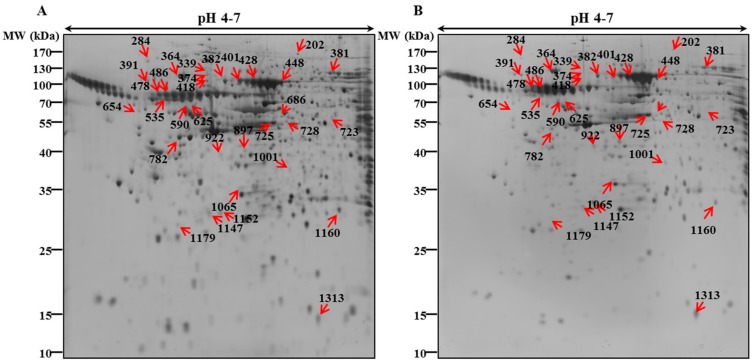
Representative Two-dimensional gel electrophoresis proteome maps of the control and pectolinarigenin-treated AGS cells. The (**A**) control and (**B**) PEC-treated (100 μM) of AGS cells, the cells were incubated with 100 µM of PEC for 24 h. The total proteins were separated on 18 cm linear IPG strips (pH 4–7) in the first dimension and in the second dimension with 12% second dimension sodium dodecyl sulfate-polyacrylamide gel electrophoresis (SDS-PAGE). The gels were silver stained. The numbered arrows indicate protein spots successfully identified by matrix-assisted laser desorption/ionization time-of-flight/time-of-flight tandem mass spectrometry. The experiments were performed in triplicate.

**Figure 3:**
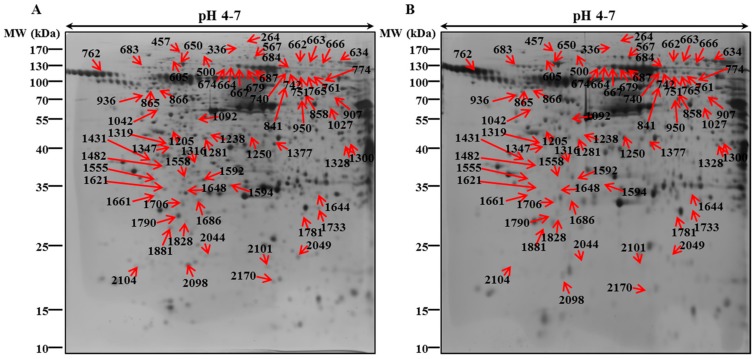
Representative Two-dimensional gel electrophoresis maps of the control and pectolinarigenin-treated MKN28 cells. The (**A**) control and (**B**) PEC-treated (100 μM) of MKN28, the cells were incubated with the 100 µM of PEC for 24 h. The total proteins were separated on 18 cm linear IPG strips (pH 4–7) in the first dimension and in the second dimension with 12% SDS-PAGE. The gels were then silver stained. The numbered arrows indicate protein spots successfully identified by matrix-assisted laser desorption/ionization time-of-flight mass spectrometry (MALDI/TOF-MS). The experiments were performed in triplicate.

**Figure 4:**
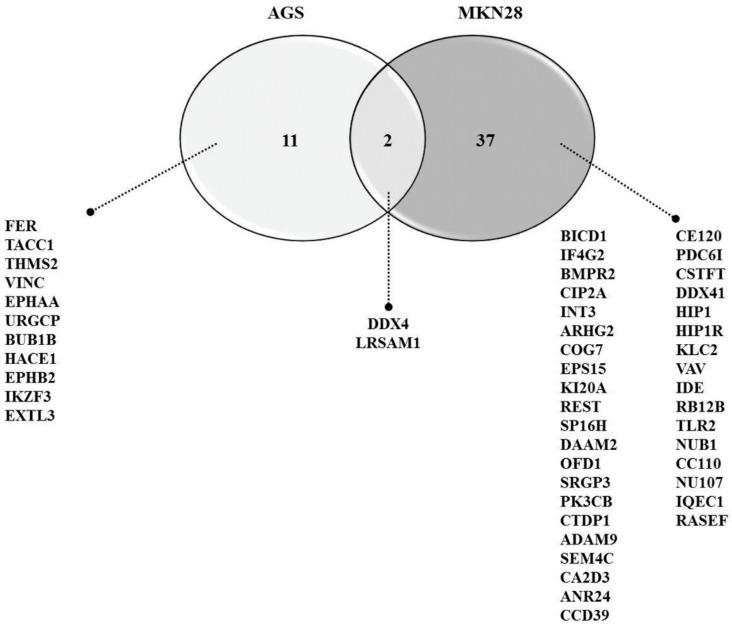
Venn diagram of differentially expressed proteins that are overlapped between AGS and MKN28 cells. A two-way Venn of AGS and MKN28 reveals two proteins that were commonly identified (DDX4, LRSAM1), while 11 and 37 proteins were uniquely identified in AGS and MKN28 cells.

**Figure 5:**
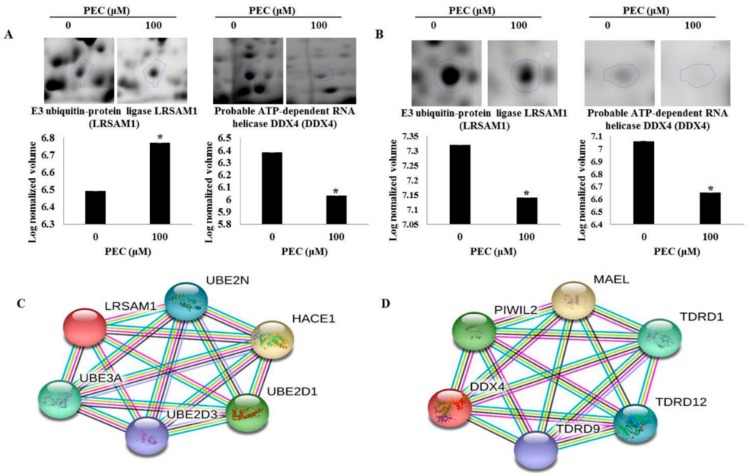
Megascopic pictures and relative volume intensity of differential expressed proteins spots identified in pectolinarigenin-treated (**A**) AGS and (**B**) MKN28 cells. Gels were scanned and image analysis was performed using Progenesis Samespots software. The spots differing significantly in their intensities (fold-change ≥ 1.5) in pectolinarigenin-treated AGS and MKN28 cells, correlated with untreated control cells. The protein (**C**) LRSAM1 and (**D**) DDX4 are interacted with other proteins in STRING database (high confidence level: 0.700, developed five signaling modules). The data are shown as the mean ± standard deviation of three independent experiments (* *p* < 0.05 vs. control).

**Figure 6:**
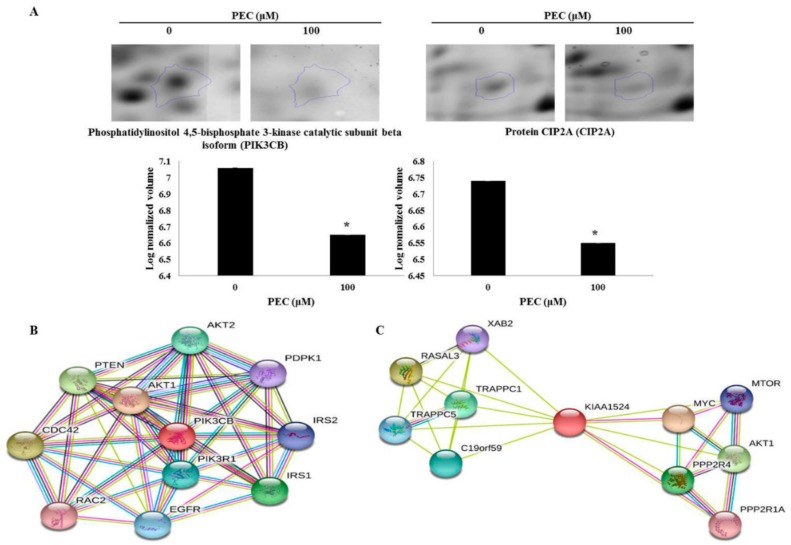
Expression profiles of magnified two-dimensional gel electrophoresis spots identified in pectolinarigenin-treated MKN28 cells. (**A**) The spots of PIK3CB and CIP2A are differentially expressed in MKN28 cells. Gels were scanned and image analysis was performed using Progenesis Samespots software. The spots differing significantly in their intensities (fold-change ≥ 1.5) in pectolinarigenin-treated AGS and MKN28 cells, as compared with untreated control cells. (**B**) PIK3CB and (**C**) CIP2A are related with PI3K/AKT/mTOR pathway in STRING database (high confidence level: 0.700 developed 10 signaling modules). The data are shown as the mean ± standard deviation of three independent experiments (* *p* < 0.05 vs. control).

In our previous study, we have investigated the mechanism underlying behind the anti-cancer effect of PEC on both AGS and MKN28 by regulating multiple cell regulations, such as cell cycle arrest, autophagy, and apoptotic cell death. We implemented 2-DE analysis by confirming the representative 2-DE patterns of the untreated (control) and PEC-treated (100 μM) AGS and MKN28 cells (Figure 2 and Figure 3). In AGS and MKN28 cells treated with PEC, a total of 29 and 56 differentially expressed protein spots were identified (Fold change ≥ 1.5; *p* < 0.05) applying Progenesis Samespots image analysis software (version 4.0.), respectively. Finally, 13 and 39 differentially expressed proteins were identified on both AGS and MKN28 cells, respectively, by using MALDI-TOF/TOF-MS analysis upon database searching. In AGS cell treated with PEC group: seven proteins were up-regulated and six were down-regulated and in case of, MKN28 cell treated with PEC group: 14 proteins were up-regulated and 25 were down-regulated. The description of all identified proteins with their corresponding Swissprot accession number, analytical molecular weight, analytical isoelectric point, sequence coverage and the number of peptide matches, MOWSE score, and fold change are shown in Table 1 and Table 2. Among the differential expressed proteins we found two proteins LRSAM1 and DDX4, which are commonly expressed in both AGS and MKN28 cells treated with PEC. Remaining 11 and 37 proteins are uniquely differentiated only in AGS and MKN cells treated with PEC respectively as shown in Figure 4. As shown in Figure 5A,B, LRSAM1 was up-regulated, and DDX4 was down-regulated in AGS cells, whereas both LRSAM1 and DDX4 were down-regulated in MKN28 cells that were treated with PEC, as compared with the control cells. The LRSAM1 protein cluster contains biological processes, such as protein polyubiquitination (GO: 0000209), protein K48-linked ubiquitination (GO: 0070936), and protein catabolic process (GO: 0030163), as shown in Figure 5C. The DDX4 cluster involves biological processes, like DNA methylation involved in gamete generation (GO: 0043046), mitotic cell cycle (GO: 0051321), and gene silencing by RNA (GO: 0031047), as shown in Figure 5D. Moreover, as shown in Figure 6A, we found PIK3CB and CIP2A another two proteins were down-regulated in PEC treated MKN28 cells as compared to the control group. The protein clusters are related with PI3K/AKT/mTOR pathway in MKN28 cells, as shown in Figure 6B,C.

### 3.3. Validation of Differentially Expressed Proteins by Western Blot Analysis.

**Figure 7:**
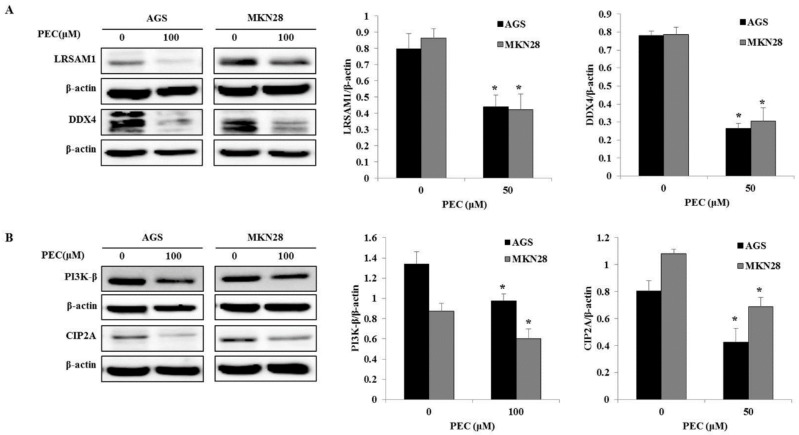
Validation of differentially expressed proteins using immunoblotting. (**A**,**B**) The cell lysates prepared from Control (DMSO)—and PEC (100 μM)-treated cells were subjected to SDS-PAGE for protein separation and LRSAM1, DDX4, PI3K-β, and CIP2A proteins were detected using the corresponding antibodies, and β-actin was used as a loading control. The experiments were performed in triplicate (* *p* < 0.05 vs. control).

Immunoblotting analysis was performed to verify the expression of these differentially expressed proteins, which were identified in the PEC-treated AGS and MKN28 cells using proteome analysis. As shown in Figure 7A,B, the western blot analysis revealed that the protein expression of LRSAM1 (LRSM1), DDX4, PI3K-β (PK3CB), and CIP2A were significantly decreased in the PEC-treated AGS and MKN28 cells, as compared with the control (*p* < 0.05). These data suggested that the results of the immunoblotting were consistent with those of the comparative proteomic analysis. On the other hand, the LRSAM1 protein of PEC-treated AGS cells showed an opposite expression from the proteomic analysis.

### 3.4. Molecular Docking Studies

**Figure 8:**
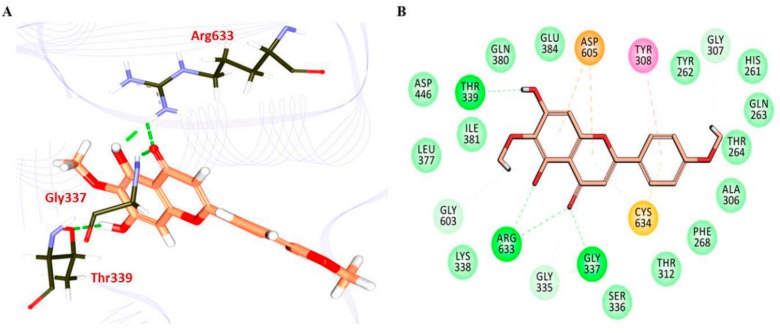
Intermolecular hydrogen bond interactions between protein and the ligands. (**A**) DDX4-Pectolinaringenin complex. (**B**) The residues Gly307, Gly335, Ser336, Lys338, and Gly603 have demonstrated the carbon-hydrogen bonds.

Molecular docking studies have revealed and confirmed that PEC ligand have interacted strongly with the protein conferred by a -Cdocker interaction energy of 43.97 kcal/mol. PEC has disclosed that the ligand has formed four hydrogen bond conferred by the residues Gly337, Thr339, and Arg633, respectively, Figure 8A. Additionally, the residues Tyr308, Asp605, and Cys634 have displayed π-π, π-anion, and π-alkyl and π-sulphur bonds in that order. The residues Gly307, Gly335, Ser336, Lys338, and Gly603 have demonstrated the carbon-hydrogen bonds, Figure 8B. Molecular docking data give as an additional confirmation to show the ability of PEC to bind with DDX4 and inhibiting its protein expression.

### 3.5. Protein-Protein Interaction

**Figure 9:**
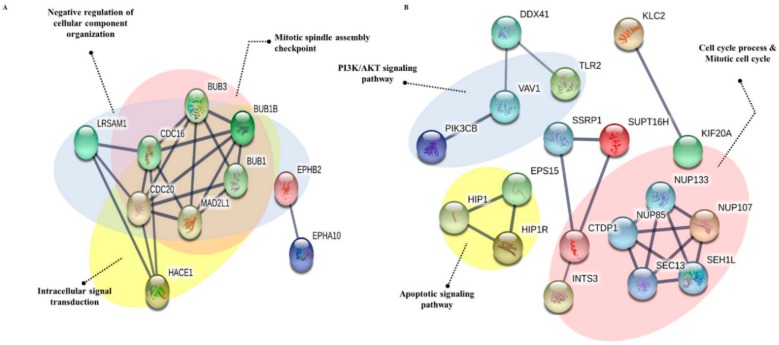
STRING analysis of significant proteins in AGS and MKN28 cells. (**A**) STRING database, version 10.5 (http://string-db.org) was used to determine the protein-protein interactions of the 13 proteins differentially expressed in AGS cells treated with PEC. (**B**) Protein-protein interactions of the 39 proteins differentially expressed in MKN28 cells treated with PEC. Interactions predicted with high confidence were included in the analyses, and proteins with no predicted interactions were removed.

Most of the differentially expressed proteins from PEC treated group are involved in cancer regulation. To anticipate protein-protein interactions and protein complexes, along with putative pathways, the above proteins were subjected to STRING analysis. STRING generated interconnected protein network and developed five signaling modules after clustering with a high confidence level 0.700. As shown in Figure 9A,B, AGS and MKN28 cells showed a differential pattern of protein interactions in PEC treated groups. AGS cells group containing LRSAM1, BUB1B, HACE1, and EPHB2 proteins formed three clusters, one is a cluster of negative regulation of cellular component organization, the other is a cluster of mitotic spindle assembly checkpoint, and the final one is a cluster of intracellular signal transduction. Whereas, the MKN28 cells group containing KIF20A, NUO107, DDX41, TLR2, VAV1, PIK3CB, and HIP1R proteins formed three clusters, one is a cluster of PI3K/AKT signaling pathway, the other is a cluster of cell cycle process and mitotic cell cycle, and the final one is a cluster of the apoptotic signaling pathway.

### 3.6. Gene Ontology Analysis

**Figure 10:**
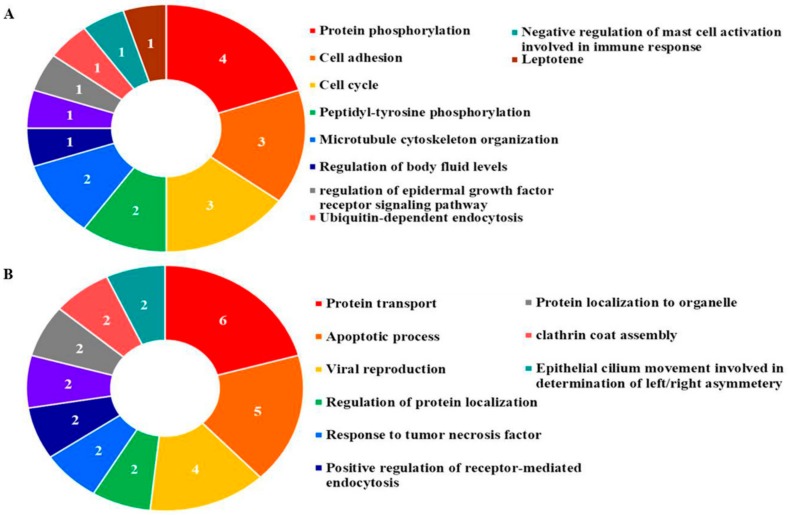
Gene ontology analysis of (**A**) AGS and (**B**) MKN28 cells. The pie charts representing the distribution of the identified proteins according to their biological process. Gene ontology analyses of the determined proteins were assigned according to their biological function, using the web-based tool at GeneCodis (http://genecodis.cnb.csic.es).

In order to understand the biological relevance of PEC regulated proteins, as shown in Figure 10A,B and Table 3, the gene ontology (GO) terms for biological processes were investigated for all identified proteins. The GO results demonstrated that the highest associations were with the biological processes regulation of the epidermal growth factor receptor signaling pathway (GO: 0042058), related cell cycle (GO: 0007049), and negative regulation of endocytosis (GO: 0045806) in PEC-treated AGS cells. Apoptotic process (GO: 0006915), M phase of mitotic cell cycle (GO: 0000087), cell death (GO: 0008219), positive regulation of receptor-mediated endocytosis (GO: 0048260), and positive regulation of macrophage fusion (GO: 0034241) in PEC-treated MKN28 cells.

## 4. Discussion

In recent times, the comparative proteomic analysis is frequently employed for the identification of changes in protein expression upon drug treatment on cancer cells. These data could furnish clues for the examination of the effects of drug and further understanding of the mechanisms at the molecular level. In this study, AGS and MKN28 gastric cancer cell lines were used as in vitro models. The MTT assay analysis confirmed the antitumor effect of PEC at the cellular level. Our current and previous data demonstrate that PEC significantly inhibited cell proliferation and induced cell cycle arrest, autophagy, and apoptosis in both the cell lines. In order to examine changes at the protein level, a proteomic approach using 2-DE coupled with mass spectrometry was undertaken to identify the altered proteins in AGS and MKN28 human gastric cancer cells in response to PEC treatment. A total of 85 (29 + 56) differentially expressed protein spots were detected in both of the cell lines, among which 52 (13 + 39) spots were successfully identified by MALDI-TOF/TOF mass spectrometry. All of the protein spots were not identified because of relatively low concentrations and also due to sensitivity limitations in mass spectrometry. In AGS cells, a total of 13 differentially expressed proteins were identified, among which seven were up-regulated and six were down-regulated. Whereas, in MKN28 a total of 39 differentially expressed proteins were identified, among which 14 were up-regulated and 25 were down-regulated. The identified proteins were predominantly involved in tumor growth and progression, cell cycle progression, autophagy and the apoptosis in gastric cancer cells. These results indicated that PEC induces inhibited cell proliferation, cell cycle arrest, autophagy and apoptotic cell death in both AGS and MKN28 cells by regulating those proteins. Of these, LRASM1 and DDX4 two proteins were altered in both the gastric cancer cell lines treated with PEC. Changes in the same proteins in different cell lines apparently symbolize the general effect of PEC against gastric cancer cells. LRASM1, E3 ubiquitin-protein ligase recognize and ubiquitinate various bacteria by initiating the autophagic reaction. This reflects that, LRSAM may play an imperative role in resistance to cellular bacteria by autophagy [21,22]. A recent finding indicates that the level of LRSAM1 is significantly up-regulated in patients with colorectal cancer, implying that the aberrant expression of LRSAM1 may be involved in the cancer progression [23]. DDX4 is another commonly modified protein in both the cell lines, which is ATP-dependent RNA helicase with proven essential roles in cell proliferation and migration, which is consistently localized with the mitotic apparatus in various blood-derived cancer cells [24,25]. Moreover, it has been reported that DDX4 is expressed in several ovarian cancer cells and tissues, and its overexpression stimulates cell cycle progression by abolishing the G2 checkpoint [26]. The immunoblotting results confirmed that the expression of LRSAM1 and DDX4 was significantly down-regulated in both AGS and MKN28 cells. Molecular docking suggest that PEC could bind with both the proteins independently, revealing the quintessential binding modes of PEC.

We also determined two crucial proteins, which are PK3CB and CIP2A down-regulated in MKN28 cells. The phosphatidylinositol-4, 5-bisphosphate 3-kinase catalytic subunit beta isoform (PK3CB or PI3K-β) is known to be associated with a diverse group of cellular functions, including cell growth, proliferation, and intracellular trafficking [27,28]. It is known that PK3CB participate in phosphoinositide 3-kinase (PI3K) pathway, which is crucial for cell growth metabolism [29]. CIP2A is a recently identified oncogene that inhibits protein phosphatase 2A (PP2A) and stabilizes c-Myc in cancer cells [30]. Protein phosphatase 2A (PP2A) is a tumor suppressor that plays an essential role in the regulation of cell homeostasis through the negative regulation of signaling pathways initiated by protein kinases. Increased expression levels of CIP2A have been reported in gastric, colon, breast, and lung cancers [31,32]. It demonstrated that normal human cells that are immortalized by overexpression of TERT and inhibition of p53 and Rb, could not be transformed by oncogenic forms of H-ras without simultaneous inhibition of PP2A activity [33]. The immunoblotting confirmation showed these two proteins were significantly decreased by PEC in both AGS and MKN28 cells. These confirmed results influence with our previous study that [15] the down-regulation PI3K/Akt/mTOR pathway leads to G2/M phase cell cycle arrest, autophagic, and apoptotic cell death in gastric cancer treated with PEC.

Bioinformatics analysis revealed that three differential expressed proteins were involved in cell cycle arrest in AGS cells (TACC1, BUB1B, and URGCP), whereas, in the case of MKN28 cells, four differential expressed proteins were involved in cell cycle arrest (KIF20A, NU107, DDX4, and LRSAM1) in PEC treated cells. The TACC1 is transforming acidic coiled-coil-containing protein 1. This protein has not yet been determined however, it is speculated that it may be represented as breast cancer candidate gene. The BUB1B is mitotic checkpoint serine/threonine-protein kinase that encodes kinase involved in spindle checkpoint operation and chromosome segregation. The protein has been localized to the kinetochore and plays a role in the inhibition of the anaphase-promoting complex/cyclosome, procrastinating the onset of anaphase and providing proper chromosome segregation. Impaired spindle checkpoint operation has been found in many forms of cancer [34,35,36]. The URGCP is up-regulated gene 4, also known as URG4. The role of URG4 in the gastric carcinogenesis still remains ambiguous. Previous research shown that the overexpression of URG4 in GES cells up-regulated cyclin D1, whereas the repression of URG4 in SGC7901 and MKN28 cells down-regulated cyclin D1 [37]. KIF20A is involved in tumor progression and angiogenesis and was previously reported to be highly expressed in various cancers, such as non-small-cell lung cancer, pancreatic cancer, bladder cancer, and cholangiocellular carcinoma [38,39,40]. The down-regulation of KIF20A was previously demonstrated to markedly suppress pancreatic cancer cell growth, indicating that KIF20A might be an oncoantigen. In our results, KIF20A was down-regulated in MKN cells that were treated with PEC. Previously, it was reported that NUP107 proteins are overexpressed in many types of cancers, including breast, prostate, colon, etc. [41]. Our results revealed the down-regulation of NUP107 in PEC treated MKN28 cells indicating the suppressive effect of PEC on nuclear pore complexes (NPCs). In addition, we found several proteins that involved in apoptotic and cell death process in MKN28 cells treated with PEC are HIP-1, VAV-1, PDC6IP, DDX41, ARHG2, and IF4G2. The hunting-interacting protein 1 (HIP-1) is a protein that interacts with the huntingtin protein and known to accommodate a domain homologous to the death effector domains found on proteins that are involved in apoptosis [42]. HIP1’s pro-apoptotic effect may associate with activation of caspase-8 and a novel HIP1 protein interactor HIPPI [43]. HIP1 has also been found to be overexpressed in some cancers, including a subgroup of colorectal and prostate cancers [44]. Vav1 functions as a signal transducer protein in the hematopoietic system, where it is comprehensively expressed. Vav1 was recently implicated in several human cancers, including neuroblastoma, lung, and pancreatic [45,46]. The programmed cell death 6-interacting protein (PDCD6IP) encodes a protein thought to participate in programmed cell death [47,48]. The rho guanine nucleotide exchange factor 2 (ARHGEF2) play an essential role in diverse cellular processes that are initiated by extracellular stimuli [48]. The eukaryotic translation initiation factor 4 gamma 2 (EIF4G2) is a cap binding protein complex that consists of three subunits, which are eIF4A, eIF4E, and eIF4G [49].

In summary, Proteomic analysis of the differentially expressed protein profiles illustrate that the differential proteins are strongly related to apoptotic process, cell cycle arrest, and tumor suppressers. Our comparative proteomic approach contributes a broad and adequate technique to identify protein expression profile in response to PEC treatment in gastric cancer cells. Identification and characterization of functionally modulated proteins that are involved in PEC-induced cellular responses will lead to a phenomenal cognizance about mechanisms underlying the anticancer effect of PEC, a flavonoid monomer, and will immensely contribute to the future clinical development of unique therapeutic drugs.

## Figures and Tables

**Table 1:** List of differentially expressed proteins in AGS cells treated with PEC, identified using MALDI-TOF/TOF-MS analysis.

No.	Spot No.	Accession No.	Protein Name	MOWSE Score	Sequence Coverage (%)/Peptides Matched	Theoretical Mr (Da)	Theoretical pI Value	Fold Change	Up/Down	*p*-Value (Anova)
1	364	P16591	Tyrosine-protein kinase Fer	1.04 × 10^6^	22.1/21	94,639	6.7	2.1	**↑**	0.001
2	478	O75410	Transforming acidic coiled-coil- containing protein 1	1,640,000	17.1/14	87,795	4.8	1.8	**↑**	0.003
3	728	Q6UWE0	E3 ubiquitin-protein ligase LRSAM1	1,510,000	23/18	83,595	5.7	1.9	**↑**	0.003
4	723	Q5TEJ8	Protein THEMIS2	7.09 × 10^6^	17.9/8	72,049	5.8	2.3	**↓**	0.008
5	725	P18206	Vinculin	1.01 × 10^6^	16.3/14	123,800	5.5	1.7	**↑**	0.009
6	1065	Q5JZY3	Ephrin type-A receptor 10	1.19 × 10^6^	16/11	109,717	6.5	2.7	**↓**	0.01
7	448	Q8TCY9	Up-regulator of cell proliferation	1.77 × 10^6^	13.7/16	104,988	6	1.6	**↑**	0.01
8	1147	O60566	Mitotic checkpoint serine/threonine-protein kinase BUB1 beta	2.57 × 10^6^	12.2/12	119,546	5.2	1.9	**↓**	0.014
9	202	Q8IYU2	E3 ubiquitin-protein ligase HACE1	1.58 × 10^6^	12.9/10	102,343	5.6	3.1	**↓**	0.027
10	339	P29323	Ephrin type-B receptor 2	1.45 × 10^6^	15/12	117,494	6.1	2.4	**↓**	0.033
11	381	Q9UKT9	Zinc finger protein Aiolos	2.12 × 10^6^	24.4/8	58,024	6.1	1.5	**↑**	0.035
12	418	Q9NQI0	Probable ATP-dependent RNA helicase DDX4	1.23 × 10^6^	15.9/9	79,308	5.6	2.2	**↓**	0.036
13	1001	O43909	Exostosin-like 3	8.19 × 10^7^	17/16	104,750	6.1	2.5	**↑**	0.047

↑ higher expression; ↓ lower expression.

**Table 2:** List of differentially expressed proteins in MKN28 cells treated with PEC, identified using MALDI-TOF/TOF-MS analysis.

No.	Spot No.	Accession No.	Protein Name	MOWSE Score	Sequence Coverage (%)/Peptides Matched	Theoretical Mr (Da)	Theoretical pI Value	Fold Change	Up/Down	*p*-Value (Anova)
1	774	Q96G01	Protein bicaudal D homolog 1	3.68 × 10^6^	15.4/16	110,751	5.6	2.1	**↑**	0.002
2	2101	P78344	Eukaryotic translation initiation factor 4 gamma 2	1.50 × 10^6^	12.2/13	102,363	6.7	2.9	**↓**	0.002
3	1482	Q13873	Bone morphogenetic protein receptor type-2	1.77 × 10^6^	12.8/11	115,202	5.8	2.5	**↓**	0.002
4	1027	Q8TCG1	Protein CIP2A	2.92 × 10^6^	15.5/13	102,186	5.9	1.5	**↓**	0.002
5	765	Q68E01	Integrator complex subunit 3	7.80 × 10^6^	16/13	118,071	5.5	7.3	**↑**	0.003
6	1238	Q92974	Rho guanine nucleotide exchange factor 2	1.39 × 10^6^	15.1/13	111,544	6.9	2.1	**↓**	0.003
7	1828	P83436	Conserved oligomeric Golgi complex subunit 7	1.84 × 10^6^	15.7/8	86,345	5.3	3.9	**↓**	0.004
8	1881	P42566	Epidermal growth factor receptor substrate 15	2.84 × 10^10^	23.4/19	98,657	4.5	2.7	**↓**	0.004
9	2049	O95235	Kinesin-like protein KIF20A	7.75 × 10^6^	16.2/12	100,279	6.5	2.3	**↓**	0.005
10	1250	Q13127	RE1-silencing transcription factor	1.88 × 10^6^	16.8/19	121,873	6.3	2.3	**↑**	0.005
11	866	Q9Y5B9	FACT complex subunit SPT16	6.78 × 10^11^	30.6/37	119,915	5.5	1.7	**↑**	0.007
12	1281	Q86T65	Disheveled-associated activator of morphogenesis 2	1.35 × 10^7^	16.8/21	123,500	6.4	1.5	**↓**	0.007
13	1477	O75665	Oral-facial-digital syndrome 1 protein	1.49 × 10^6^	15.7/21	116,672	5.8	2.1	**↓**	0.008
14	650	O43295	SLIT-ROBO Rho GTPase-activating protein 3	2.74 × 10^9^	22.2/26	124,505	6.2	1.8	**↑**	0.008
15	1558	P42338	Phosphatidylinositol 4,5-bisphosphate 3-kinase catalytic subunit beta isoform	3.92 × 10^6^	15.7/15	122,763	6.7	2.6	**↓**	0.008
16	1319	Q9Y5B0	RNA polymerase II subunit A C-terminal domain phosphatase	4.51 × 10^6^	17/13	104,400	5.2	2.4	**↓**	0.009
17	1431	Q13443	Disintegrin and metalloproteinase domain-containing protein 9	3.67 × 10^6^	17.6/12	90,557	7.7	2.2	**↓**	0.009
18	1648	Q6UWE0	E3 ubiquitin-protein ligase LRSAM1	1.48 × 10^6^	16.3/14	83,595	5.7	1.5	**↓**	0.009
19	663	Q9C0C4	Semaphorin-4C	368,935	13.2/8	92,624	6.9	2.7	**↑**	0.009
20	605	Q8IZS8	Voltage-dependent calcium channel subunit alpha-2/delta-3	1.19 × 10^6^	16.5/15	123,012	5.5	2.6	**↓**	0.01
21	500	Q8TF21	Ankyrin repeat domain-containing protein 24	1.88 × 10^6^	14/15	124,188	5	2.5	**↑**	0.01
22	1706	Q9UFE4	Coiled-coil domain-containing protein 39	5.24 × 10^6^	19.4/19	109,901	6.1	2.2	**↓**	0.011
23	2098	Q8N960	Centrosomal protein of 120 kDa	2.94 × 10^6^	19/19	112,641	5.9	2.3	**↓**	0.011
24	907	Q8WUM4	Programmed cell death 6-interacting protein	1.07 × 10^6^	15/12	96,024	6.1	1.8	**↑**	0.012
25	336	Q9H0L4	Cleavage stimulation factor subunit 2 tau variant	7.95 × 10^6^	21.1/18	64,437	6.8	1.6	**↓**	0.013
26	841	Q9UJV9	Probable ATP-dependent RNA helicase DDX41	1.14 × 10^6^	16.7/15	69,838	6.4	1.9	**↑**	0.013
27	858	O00291	Huntingtin-interacting protein 1	1.99 × 10^6^	20.3/25	116,222	5.2	3	**↑**	0.015
28	2170	Q9H0B6	Kinesin light chain 2	6.75 × 10^6^	22.3/13	68,935	6.7	2.3	**↓**	0.021
29	634	P15498	Proto-oncogene vav	3.27 × 10^6^	16/13	98,315	6.2	2.2	**↑**	0.023
30	1790	P14735	Insulin-degrading enzyme	8.77 × 10^11^	22.1/28	117,970	6.2	1.6	**↓**	0.023
31	751	Q8IXT5	RNA-binding protein 12B	1.05 × 10^6^	16.4/15	118,104	6.3	1.7	**↑**	0.025
32	1644	O60603	Toll-like receptor 2	1.03 × 10^6^	16.8/13	89,838	6.2	1.8	**↓**	0.027
33	2044	Q9NQI0	Probable ATP-dependent RNA helicase DDX4	1.06 × 10^7^	18.2/13	79,308	5.6	2.6	**↓**	0.032
34	831	Q9Y5A7	NEDD8 ultimate buster 1	5.37 × 10^6^	20.7/15	70,539	5.7	2.4	**↑**	0.035
35	1377	Q8TBZ0	Coiled-coil domain-containing protein 110	1.97 × 10^6^	19.9/21	96,726	5.9	1.6	**↓**	0.035
36	1562	P57740	Nuclear pore complex protein Nup107	3.63 × 10^6^	18.9/15	106,375	5.3	1.5	**↓**	0.036
37	1686	O75146	Huntingtin-interacting protein 1-related protein	1.39 × 10^6^	11.1/13	119,389	6.2	2.5	**↓**	0.038
38	1340	Q6DN90	IQ motif and SEC7 domain-containing protein 1	3.48 × 10^6^	15.7/16	108,315	6.5	2.3	**↓**	0.042
39	664	Q8IZ41	Ras and EF-hand domain-containing protein	1.35 × 10^6^	18/12	82,880	5	2.3	**↑**	0.043

↑ higher expression; ↓ lower expression.

**Table 3:** List of tumor-associated biological process of differentially expressed genes in AGS and MKN28 cells.

Cell Line	GO Accession	Biological Process	Number of Genes	Gene Symbol	Gene Name	*p*-Value
**AGS Cells**	GO: 0042058	Regulation of epidermal growth factor receptor signaling pathway	1	FER	Tyrosine-protein kinase Fer	1.14 × 10^−3^
	GO: 0007049	Cell Cycle	3	TACC1	Transforming acidic coiled-coil-containing protein 1	5.31 × 10^−4^
				BUB1B	Mitotic checkpoint serine/threonine-protein kinase BUB1 beta	
				URGCP	Up-regulator of cell proliferation	
	GO: 0045806	Negative regulation of endocytosis	1	LRSAM1	E3-ubiquitin-protein ligase	3.42 × 10^−3^
**MKN28 Cells**	GO: 0006915	Apoptotic process	5	HIP1	Huntingtin-interacting protein 1	4.85 × 10^−4^
				VAV	Proto-oncogene vav	
				PDC6I	Programmed cell death 6-interacting protein	
				DDX41	Probabale ATP-dependnet RNA helicase DDX41	
				ARHG2	Rho guanine nucleotide exchange factor 2	
	GO: 0000087	M phase of mitotic cycle	2	KI20A	Kinesin-like protein KIFA20A	5.03 × 10^−3^
				NU107	Nuclear pore complex protein Nup107	
	GO: 0008219	Cell death	2	HIP1	Huntingtin-interacting protein 1	1.30 × 10^−3^
				IF4G2	Eukaryotic translation initiation factor 4 gamma 2	
	GO: 0048260	Positive regulation of receptor-mediated endocytosis	2	HIP1	Huntingtin-interacting protein 1	1.08 × 10^−4^
				BICD1	Protein bicaudal D homolog 1	
	GO: 0034241	Positive regulation of macrophage fusion	1	ADAM9	Disintegrin and metalloproteinase domain-containing protein 9	1.11 × 10^−3^
